# Concise synthesis of artemisinin from a farnesyl diphosphate analogue

**DOI:** 10.1016/j.bmc.2017.03.068

**Published:** 2018-04-01

**Authors:** Xiaoping Tang, Melodi Demiray, Thomas Wirth, Rudolf K. Allemann

**Affiliations:** School of Chemistry, Cardiff University, Park Place, Main Building, Cardiff CF10 3AT, UK

**Keywords:** Malaria, Artemisinin, Amorphadiene synthase, Dihydroartemisinic aldehyde, Dihydroartemisinic acid, Farnesyl diphosphate

## Abstract

Artemisinin is one of the most potent anti-malaria drugs and many often-lengthy routes have been developed for its synthesis. Amorphadiene synthase, a key enzyme in the biosynthetic pathway of artemisinin, is able to convert an oxygenated farnesyl diphosphate analogue directly to dihydroartemisinic aldehyde, which can be converted to artemisinin in only four chemical steps, resulting in an efficient synthetic route to the anti-malaria drug.

## Graphical abstract

**Figure:**
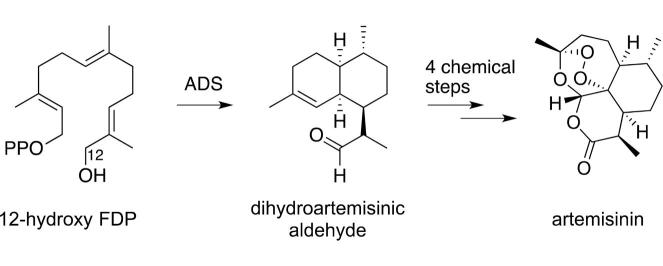

## 1 Introduction

Malaria affects almost 50% of the world’s population and causes hundreds of thousands of deaths each year.^1^ Isolated from the plant *Artemisia annua* (qinghaosu), the sesquiterpene artemisinin (**1**) exhibits excellent anti-malaria activity and kills the parasite at most of its asexual stages of development in human blood.^2^ Artemisinin-based combination treatments (ACTs) are widely used as the first-line treatment for malaria.^3^ Although several synthetic routes to artemisinin (**1**) have been developed,^4^ the chemical synthesis is lengthy and low yielding due to the highly complex structure of the sesquiterpene endoperoxide. The worldwide supply of artemisinin (**1**) relies predominantly on the extraction of (**1**) from the plant *Artemisia annua*^5^ and as a consequence the world market price is highly volatile ranging from US $350 to $1700 per kilogram.^6^ Most countries affected by malaria epidemics are in the developing world, and therefore a stable and affordable supply of artemisinin (**1**) is highly desirable.

**Scheme 1:**
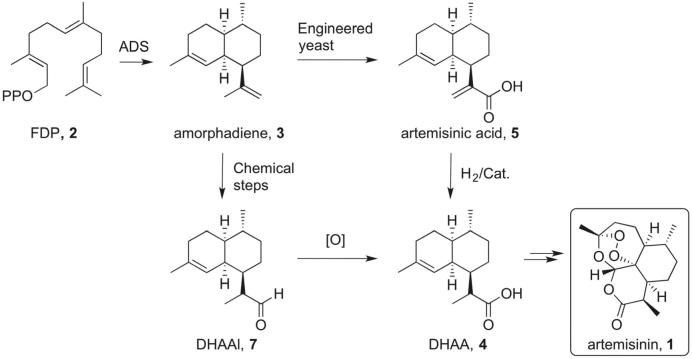
Synthesis of artemisinin (**1**) from farnesyl diphosphate (**2**).

Currently the most efficient synthetic route to produce artemisinin is the combination of a biosynthetic process with several chemical steps (Scheme 1). The biosynthesis of artemisinin is well understood^7^ and the key step to this synthesis involves the class I sesquiterpene cyclase amorphadiene synthase (ADS). This enzyme catalyses the cyclisation of (*E*,*E*)-farnesyl diphosphate (FDP, **2**) to amorpha-4-11-diene (**3**), a bicyclic intermediate with four stereocentres. **3** can be converted to the advanced synthetic intermediate dihydroartemisinic acid (DHAA, **4**) either chemically8a or enzymatically using engineered yeast (Scheme 1).(b), (c) The latter method has been developed into a semi-synthetic production of artemisinin (**1**). Engineered yeast containing ADS and five other enzymes produce artemisinic acid (**5**), which is subsequently reduced to DHAA (**4**) by a transition metal-catalysed hydrogenation.(a), 9 DHAA (**4**) can then be converted to artemisinin (**1**) in three well-established steps.^10^ The pharmaceutical company Sanofi developed a commercial route for biosynthetically produced artemisinin in 2014, but this process has now discontinued due to strong market forces.^11^ Alternative routes for the low-cost production of artemisinin (**1**) are therefore urgently required.

**Scheme 2:**
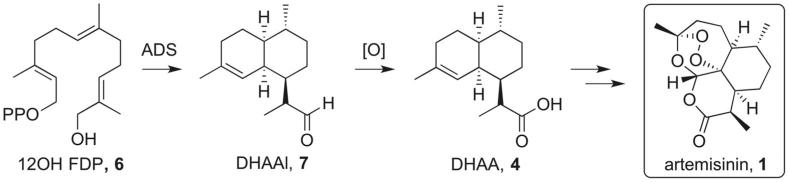
Schematic synthesis of artemisinin (**1**) from 12-hydroxyfarnesyl diphosphate (**6**).

Here we report a novel synthetic route to artemisinin (**1**) starting from the oxygenated farnesyl diphosphate analogue 12-hydroxyfarnesyl diphosphate (**6**) (Scheme 2). Amorphadiene synthase (ADS) is able to convert **6** in a single step to dihydroartemisinic aldehyde (DHAAl, **7**), an advanced intermediate of artemisinin.^12^ This route does not proceed *via* amorphadiene (**3**) and therefore avoids several redox steps. Increasing the oxidation state at the linear precursor stage produces a two-step synthesis of **4**, which significantly shortens the synthesis of artemisinin (**1**).

## 2 Enzymatic reaction

**Scheme 3:**
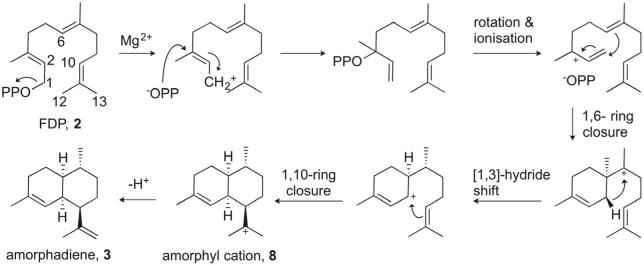
Biosynthetic pathway to amorpha-4,11-diene (**3**).

ADS catalyses the Mg^2+^-dependent, highly chemo- and stereoselective cyclisation of FDP (**2**) to amorphadiene (**3**) (Scheme 3).^13^ ADS cleaves the C—O bond in **2** and generates diphosphate and a carbocation, which rearranges through a series of ring closures and hydride transfer processes. The last step in the enzymatic sequence is the deprotonation of amorphyl cation (**8**) to yield amorphadiene (**3**) (Scheme 3).

**Scheme 4:**
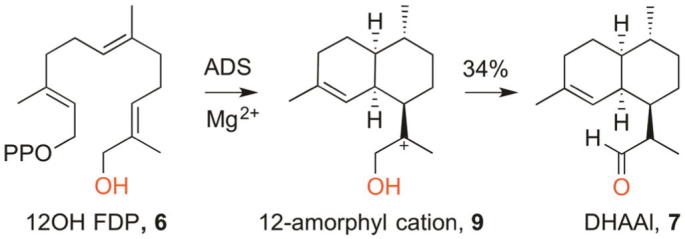
ADS-catalysed cyclisation of 12-hydroxyfarnesyl diphosphate (**7**).

The enzyme’s remarkable ability to convert a linear precursor to a structurally and stereochemically complex cyclic product offers a very efficient synthetic route to terpenes. Sesquiterpene synthases are not only highly effective and often stereospecific in the reactions they catalyse, many of them also display some degree of substrate promiscuity and some analogues of FDP (**2**) can be converted to modified terpenoids.^14^ As an example, it has been shown that ADS catalyses the cyclisation of 12-hydroxy FDP (**6**) to produce dihydroartemisinic aldehyde (**7**) (Scheme 4) with a 34% yield.^12^ The seemingly moderate yield is common for sesquiterpene cyclases as the reaction is limited by the release of the hydrocarbon products from the aqueous incubation media.^15^ Aldehyde **7** is a well-established intermediate in the biosynthesis of artemisinin (**1**).^7^ In contrast to the three redox steps required to convert (**3**) to (**7**), consisting of an allylic oxidation of amorphadiene to dihydroartemisinic alcohol, further oxidation to the corresponding aldehyde and a final reduction to dihydroartemisinic aldehyde,9c our approach yields aldehyde **7** in a single step starting from a linear FDP precursor.

## 3 Synthesis of 12-hydroxyfarnesyl diphosphate (**7**)

**Scheme 5:**
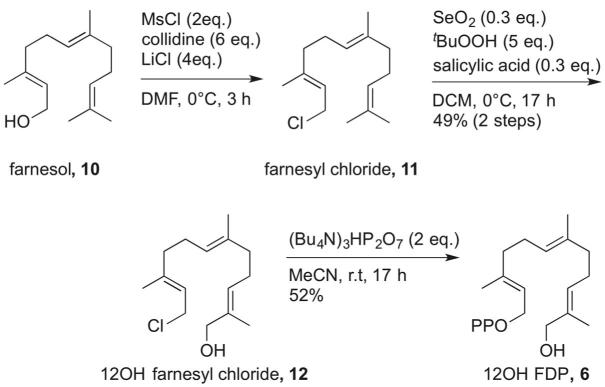
Synthesis of 12-hydroxyfarnesyl diphosphate (**6**).

12-Hydroxyfarnesyl diphosphate (**6**) was synthesised in three steps starting from commercially available (*E*,*E*)-farnesol (**10**) (Scheme 5). Chlorination of **10** gave farnesyl chloride (**11**) in a quantitative yield, which was carried forward without purification. The following step was a selenium dioxide-catalysed oxidation at C12 of **11**.^16^ The reaction conditions for the allylic oxidation were optimised, but the yield was still moderate due to the instability of the product 12-hydroxy farnesyl chloride (**12**) and the formation of a by-product resulting from the allylic oxidation at C8. Compound **12** was finally diphosphorylated^17^ under standard conditions to afford **6**.

## 4 Synthesis of artemisinin

The single step production of **7** provided a great opportunity to shorten the synthesis of artemisinin. The key intermediate DHAA (**4**) is the starting point for many syntheses developed for artemisinin (**1**).^18^ The conversion of **4** to **1** can be achieved by reaction with singlet oxygen followed by air oxidation.^10^, ^11^ The commercial route developed by Sanofi used engineered yeast to produce artemisinic acid (**5**),^9^ which was then hydrogenated with a transition metal catalyst to yield DHAA (**4**). Seeberger et al. developed a continuous flow process to convert DHAA (**4**) to artemisinin (**1**) obtaining **4** from the plant *Artemisia annua*.^10^

**Scheme 6:**
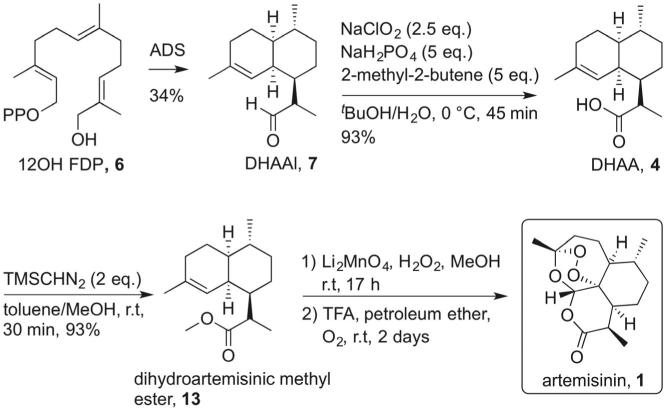
Synthesis of artemisinin (**1**) from 12-hydroxyfarnesyl diphosphate (**6**).

In our approach, DHAA (**4**) was obtained from the enzyme-produced aldehyde **7** by a simple oxidation with sodium chlorite4b in 93% yield (Scheme 6). Acid **4** was then converted to dihydroartemisinic methyl ester (**13**) with trimethylsilyldiazomethane in 94% yield.^19^ The final stage for the synthesis of artemisinin is a singlet oxygen oxidation of **13** by lithium molybdate-catalysed disproportion of hydrogen peroxide.^20^, (a) The crude product from the singlet oxygen oxidation step was taken into a hydrocarbon solvent in the presence of trifluoroacetic acid and pure oxygen.^21^ After two days, artemisinin (**1**) was formed as evidenced by NMR. At this stage, the epimeric mixture can be separated by standard column chromatography. An isolated yield cannot be given due to the small scale of this reaction. Work is on going to evaluate biological activity of the unnatural epimer.

## 5 Conclusion

A novel concise synthetic route to artemisinin (**1**) was developed. The process benefits from a new chemoenzymatic reaction between amorphadiene synthase and 12-hydroxyfarnesyl diphosphate (**6**). Due to its relaxed substrate selectivity, ADS accepts the oxygenated FDP analogue **6** to generate dihydroartemisinic aldehyde (**7**), which can be converted to artemisinin (**1**) in four steps. Different from any known synthetic route for artemisinin (**1**), this approach exploits the promiscuity of terpene synthases. Oxidation of FDP prior to cyclisation allows the ADS catalysed formation of a much-advanced intermediate on the pathway to artemisinin. The whole process only utilised one enzyme combined with known chemistry. This new route may have potential to be developed into a low-cost supply of this important antimalarial drug.

## 6 Experimental section

### 6.1 General remarks

All chemicals were purchased from Sigma-Aldrich, Acros Chemicals, Fluorochem or Alfa Aesar and used without further purification unless otherwise stated. Anhydrous acetonitrile was obtained from a MBraun SPS800 solvent purification system unless otherwise stated. Flash column chromatography was performed using Biotage Flash Purification system. ^1^H NMR and ^13^C NMR spectra were measured on a Bruker Avance 500 NMR spectrometer and Bruker Avance DPX400 spectrometer. ^1^H NMR and ^13^C NMR spectra are reported as chemical shifts in ppm downfield from TMS and J values are given in Hertz. ^31^P NMR spectra were recorded on Bruker Avance DPX400 spectrometer or Bruker Avance 500 NMR spectrometer and are reported in chemical shifts downfield from 85% H_3_PO_4_. Reverse phase HPLC was performed on a system comprising of a Dionex P680 pump and a Dionex UVD170U detector unit.

### 6.2 Preparation of ADS

#### 6.2.1 General methods

LB media was prepared by dissolving tryptone (10 g), yeast extract (5 g) purchased from Fluka and NaCl (10 g) in 1 L of deionised water. Cell lysis buffer for ADS was prepared by dissolving trizma-HCl (50 mM), NaCl (500 mM), 2-mercaptoethanol (20 mM) and glycerol (10% v/v) in deionised water. The final pH was adjusted to 8.0. Dialysis buffer for ADS was prepared by dissolving HEPES (25 mM), NaCl (100 mM) and dithiothreitol (1 mM) in deionised water. The final pH was adjusted to 7.5.

#### 6.2.2 Transformation of *E. coli* BL21 with cDNA for wild-type ADS

Cloning of the ADS gene into pET21d plasmid. The gene coding for amorphadiene synthase (ADS) from *Artemisia annua* was obtained from gene bank (JF951730). It was supplied in a pTrc99a vector between the NcoI and BamHI restriction sites (pTrc-ADS). pET21d and pTrc-ADS were digested with the endonucleases NcoI and BamHI (0.1 μL of each enzyme, 1 μL of buffer, 10 μL of plasmid, 1 h, 37 °C) and the fragments ligated using T4 DNA ligase (1:2 M ratio of pET: ADS, 0.1 μL enzyme, 2 μL of buffer, H_2_O to make total volume to 20 μL) to give a new plasmid pET21d-ADS. Supercompetent *E. coli* XL1-blue cells were transformed with 5 µL of ligated DNA and stored on ice (30 min) before being heat shocked in a water bath at 40 °C for 40 s and placed on ice for 2 min. LB medium (1 mL) was added and the solution shaken for 60 min (37 °C, 150 rpm). The cells were harvested by centrifugation (3400*g*, 1 min) and spread on an agar plate containing ampicillin (100 μg/mL) after resuspending in a minimum amount of buffer. Plates were incubated overnight at 37 °C and then stored at 4 °C. A single colony from the agar plate was used to inoculate 15 mL of LB medium containing ampicillin (100 μg/mL). The culture was incubated overnight (37 °C) and the following day centrifuged (3220*g*, 8 min). The pellet was purified using a QIAprep Spin Miniprep Kit (QIAprep Miniprep Handbook-2005). The resulting 50 μL DNA solution was stored at −20 °C. The sequence was confirmed by DNA sequence analysis (Eurofin).

*E. coli* BL21 competent cells (stored at −80 °C) were slowly defrosted in ice. Vector containing a cDNA for ADS and resistance for ampicillin (1 μL) was added to the cells. After leaving on ice for 20 min, the mixture was thermally shocked in a water bath at 40 °C for 35 s and returned to ice for 2 min. LB media (1 mL, sterilised) was added to the transformed cells under flame and the solution was shaken (150 rpm) at 37 °C for 1 h. The cells were separated from the media by centrifuging the mixture (6000 rpm) for 1 min. The cells were re-suspended in the minimum amount of LB media and the mixture was spread in an ampicillin-agar plate under flame. The plate was incubated at 37 °C for 12 h.

#### 6.2.3 Overexpression of ADS

The overnight culture was prepared the same way as AS.15b The resulting mixture was incubated at 37 °C and the growth of bacteria was monitored by checking the OD of media at 600 nm until it reached 0.5. The culture was induced by isopropyl-1-thio-β-d-galactopyranoside (60 mg). The induced culture was incubated at 20 °C for 6 h. The solutions were centrifuged at 5000 rpm for 20 min, the supernatant was discarded and the pellets were stored at −20 °C.

#### 6.2.4 Purification of ADS

All the buffers for ADS purification were cooled on ice before use. The pellets were defrosted on ice and re-suspended in cell lysis buffer (40 mL). After adding lysozyme (20 mg), the mixture was stirred at 4 °C for 30 min. The solution was sonicated in an ice bath (3 min with 5 s on/10 s off cycles) then centrifuged at 5000 rpm for 10 min. The supernatant was loaded onto a Ni^2+^ NTA column and eluted with a gradient of imidazole in cell lysis buffer (5 mM to 300 mM). The protein eluted at 100 mM imidazole. The presence of protein was confirmed by SDS-PAGE electrophoresis. All the fractions with protein were combined and dialyzed in dialysis buffer at 4 °C for 24 h. The resulting protein solution was concentrated to 10 mL final volume. The concentration of ADS was determined by Bradford Assay.^22^

### 6.3 Synthesis of 12-OH FDP (**7**)

#### 6.3.1 Farnesyl chloride (**11**)

Farnesol (**10**) (2.0 mL, 8 mmol) was dissolved in anhydrous DMF (80 mL) and cooled to 0 °C. Collidine (6.3 mL, 48 mmol) and MsCl (1.3 mL, 16 mmol) were added to the mixture. After stirring at 0 °C for 15 min, anhydrous LiCl (1.35 g, 32 mmol) was added. The reaction was stirred at 0 °C for 3 h. H_2_O (80 mL) was added and the mixture was extracted with hexane (50 mL × 3). The combined organic phases were washed with CuSO_4_ (sat.), NaHCO_3_ (sat.) and brine. The resulting solution was dried over anhydrous Na_2_SO_4_ and concentrated under reduced pressure. Compound **11** was obtained as a yellow oil and used in the next step without purification. ^1^H NMR (300 MHz, CDCl_3_): *δ* (ppm) 5.46 (1H, t, *J* = 8.0), 5.12–5.08 (2H, m), 4.12 (2H, d, *J* = 8.0), 2.23–1.98 (8H, m), 1.73 (3H, s), 1.66 (3H, s), 1.61 (6H, s).

#### 6.3.2 12-Hydroxyfarnesyl chloride (**12**)

SeO_2_ (226 mg, 2.4 mmol), salicylic acid (331 mg, 2.4 mmol) and *t*BuOOH (70%, 5.5 mL, 40 mmol) were dissolved in CH_2_Cl_2_ (40 mL) and stirred for 30 min at room temperature. The reaction mixture was then cooled to 0 °C and **11** (1.92 g, 8 mmol) in CH_2_Cl_2_ (20 mL) was added and stirred at 0 °C for 17 h. The reaction was quenched with Na_2_SO_3_ (sat.) at 0 °C. The mixture was extracted with Et_2_O (50 mL × 3). The combined organic phases were dried over anhydrous Na_2_SO_4_ and concentrated under reduced pressure. The crude oil was purified by flash chromatography on silica gel (CH_2_Cl_2_ with 1% NEt_3_), the eluent used for the column chromatography was cooled on ice before use and fractions were kept on ice. Compound **12** was obtained as a yellow oil (1.02 g, 49% over two steps). ^1^H NMR (300 MHz, CDCl_3_): *δ* (ppm) 5.46–5.40 (1H, m), 5.41–5.36 (1H, m) 5.10 (1H, m), 4.10 (2H, d, *J* = 8.0), 3.99 (2H, s), 2.16–1.99 (8H, m), 1.73 (3H, s), 1.66 (3H, s), 1.60 (3H, s); ^13^C NMR (75 MHz, CDCl_3_): *δ* (ppm) 142.7, 135.3, 134.7, 126.0, 123.7, 120.3, 69.0, 41.2, 39.4, 39.3, 26.2, 26.1, 16.1, 16.0, 13.7. HRMS (ES^+^): calcd for C_15_H_25_OCl[Na]^+^: 279.1483, found: 279.1492.

#### 6.3.3 12-Hydroxyfarnesyl diphosphate (**6**)

**12** (102 mg, 0.4 mmol) and (Bu_4_N)_3_HP_2_O_7_ (720 mg, 0.8 mmol) were dissolved in anhydrous MeCN (10 mL). The reaction was stirred at room temperature for 17 h. After removing solvent under reduced pressure, the crude oil was loaded onto an ion-exchange resin DOWEX 40-W, which was received from Aldrich in H^+^ form. The resin was converted into NH_4_^+^ form by washing with concentrated NH_4_OH, followed by deionised water until the pH dropped to 7 and finally equilibrated with ion-exchange buffer (25 mM NH_4_HCO_3_ containing 2% i-PrOH). The fractions were collected and lyophilized for 18–24 h. the resulting off-yellow solid was purified by reverse phase column chromatography (Biotage KP-C18-HS 12 g column, CV = 15 mL, H_2_O/MeCN, 0% to 5% MeCN over 10 CV, 10% to 90% MeCN over 5 CV, 90% MeCN for 5 CV, UV collection 210 nm & 220 nm). The fractions were collected and lyophilized for 18–24 h to give compound **6** as a light white powder (94 mg, 52% yield). M.p. 132–136 °C. ^1^H NMR (500 MHz, D_2_O): *δ* (ppm) 5.41–5.33 (2H, m), 5.15 (1H, t, *J* = 6.5), 4.40 (2H, t, *J* = 6.5), 3.88 (2H, s), 2.16–1.96 (8H, m), 1.65 (3H, s), 1.57 (3H, s) and 1.56 (3H, s); ^31^P NMR (121 MHz, D_2_O): *δ* (ppm) −7.93, −7.53. HRMS (ES^−^): calculated for C_15_H_25_O_8_P_2_[H][Na]^−^: 419.1001, found: 419.0983.

### 6.4 Synthesis of artemisinin (**1**)

#### 6.4.1 Dihydroartemisinic aldehyde (**7**)

Purified ADS (8.8 mL of 90 µM, 2 µM) and **6** (72 mg, 0.4 mM) were added to incubation buffer (400 mL, pH = 9.4, Glycine/NaOH) containing MgCl_2_ (190 mg, 5 mM). The mixture was overlaid with pentane (600 mL). The resulting two phased solution was slowly stirred at 4 °C for 2 days. The pentane phase was separated, dried over anhydrous Na_2_SO_4_ and concentrated under reduced pressure. The crude was purified by flash chromatography (Biotage SNAP Ultra 10 g, CV = 15 mL, Pet Ether/Et_2_O, 0% to 10% Et_2_O over 10 CV, 10% to 90% Et_2_O over 5 CV, 90% Et_2_O for 5 CV, UV collection 210 nm & 220 nm). The fractions were collected and concentrated under reduced pressure. Pure compound **7** were obtained as colourless oil (12 mg, 34% yield). Compound **7** was isolated as a mixture of epimers at C11. Ratio of the two epimers were 5:1 with (11*S*)-**7** as the major product. (11*S*-**7**): ^1^H NMR (500 MHz, CDCl_3_): *δ* (ppm) 9.62 (1H, d, *J* = 3.5), 5.26 (1H, bs), 2.48 (1H, m), 2.39 (1H, m), 1.91–1.25 (11H, m), 1.63 (3H, s), 1.08 (3H, d, *J* = 7.0), 0.87 (3H, d, *J* = 6.5). (11*R*-**7**): ^1^H NMR (500 MHz, CDCl_3_): *δ* (ppm) 9.57 (1H, d, *J* = 3.5), 5.12 (1H, bs), 2.48 (2H, m), 1.91–1.25 (11H, m), 1.63 (3H, s), 1.08 (3H, d, *J* = 7.0), 1.06 (3H, d, *J* = 7.0), 0.87 (3H, d, *J* = 6.5).

#### 6.4.2 Dihydroartemisinic acid (**4**)

Dihydroartemisinic aldehyde (9 mg, 0.04 mmol) was dissolved in a mixture of *t*-BuOH (0.4 mL) and 2-methyl-2-butene (0.05 mL) and cooled down to 0 °C. A solution of NaClO_2_ (22 mg, 0.24 mmol) and NaH_2_PO_4_ (48 mg, 0.40 mmol) in deionised water (0.25 mL) was added and the mixture was stirred at 0 °C for 45 min. The reaction mixture was diluted with diethyl ether and extracted with additional diethyl ether (3 × 5 mL). The combined organic phases were washed with brine, dried over anhydrous Na_2_SO_4_ and concentrated under reduced pressure. The crude mixture was purified by flash chromatography on silica (diethyl ether/petroleum ether: 1:4 to 1:1) to give dihydroartemisinic acid **4** as colourless oil (9 mg, 93% yield). (11*S*-**4**): ^1^H NMR (300 MHz, CDCl_3_): *δ* (ppm) 5.29 (1H, bs), 2.58–2.50 (1H, m), 2.41 (1H, bs), 1.91–1.78 (3H, m), 1.75–1.54 (3H, m), 1.42 (1H, bs), 1.25–1.17 (2H, m), 1.00–0.92 (2H, m), 1.63 (3H, s), 1.23 (3H, d, *J* = 8.0), 0.87 (3H, d, *J* = 6.5); ^13^C NMR (125 MHz, CDCl_3_): *δ* (ppm) 182.2, 135.5, 120.6, 45.0, 41.8, 39.4, 35.6, 29.9, 27.8, 26.7, 25.7, 24.9, 24.1, 19.9, 16.4; (11*R*-**4**): ^1^H NMR (500 MHz, CDCl_3_): *δ* (ppm) 5.12 (1H, bs), 2.58–2.50 (2H, m), 1.91–1.78 (3H, m), 1.75–1.54 (3H, m), 1.42 (1H, bs), 1.25–1.17 (2H, m), 1.00–0.92 (2H, m), 1.63 (3H, s), 1.21 (3H, d, *J* = 7.0), 0.87 (3H, d, *J* = 6.5); ^13^C NMR (125 MHz, CDCl_3_): *δ* (ppm) 183.7, 136.2, 119.5, 43.8, 41.9, 41.6, 36.6, 35.4, 27.9, 27.6, 26.8, 25.9, 24.0, 19.9, 15.3.

#### 6.4.3 Dihydroartemisinic methyl ester (**13**)

Acid **4** (6 mg, 0.025 mmol) was dissolved in a toluene (0.30 mL) and methanol (0.20 mL) mixture. TMSCHN_2_ (0.025 mL, 2.0 M in Et_2_O) was added and the mixture was left to stir at room temperature for 30 min. The mixture was diluted with diethyl ether, quenched with AcOH (10%) and extracted with additional Et_2_O (3 × 5 mL). The combined organic phases were washed with brine, dried over anhydrous Na_2_SO_4_ and concentrated under reduced pressure. The crude was purified by flash chromatography on silica (diethyl ether/petroleum ether: 1:9 to 1:4) to give compound **13** as colourless oil (6 mg, 94% yield). (11*S*-**13**): ^1^H NMR (500 MHz, CDCl_3_): *δ* (ppm) 5.27 (1H, bs), 3.73 (3H, s), 2.54–2.49 (1H, m), 2.24 (1H, br), 1.89–1.74 (3H, m), 1.62 (3H, s), 1.66–1.55 (3H, m), 1.51–1.43 (3H, m), 1.16 (3H, br), 0.95–0.93 (2H, m), 0.86 (3H, br); ^13^C NMR (125 MHz, CDCl_3_): *δ* (ppm) 177.4, 135.0, 120.4, 51.4, 45.1, 41.6, 39.2, 35.4, 30.3, 27.5, 26.5, 25.5, 24.8, 23.8, 19.7, 16.2; (11*R*-**13**): ^1^H NMR (500 MHz, CDCl_3_): *δ* 5.12 (1H, bs), 3.73 (3H, s), 2.54–2.49 (2H, m), 1.89–1.74 (3H, m), 1.62 (3H, s), 1.66–1.55 (3H, m), 1.51–1.43 (3H, m), 1.16 (3H, br), 0.95–0.93 (2H, m), 0.86 (3H, br); ^13^C NMR (125 MHz, CDCl_3_): *δ* (ppm) 178.2, 125.5, 119.4, 51.4, 43.9, 42.1, 41.7, 36.4, 35.2, 27.6, 27.4, 26.6, 25.7, 23.8, 19.7, 15.1.

#### 6.4.4 Artemisinin (**1**)

**13** (25 mg, 0.1 mmol) and Li_2_MoO_4_ (10 mg, 0.06 mmol) were suspended in MeOH (1 mL). H_2_O_2_ (50%, 200 µL) was added drop-wise and it was observed that the solution turned dark brown. The mixture was stirred at room temperature for 17 h. The mixture was diluted with CH_2_Cl_2_ (2 mL) and H_2_O (2 mL), the organic layer was separated and the aqueous phase was extracted with CH_2_Cl_2_ (3 × 2 mL). Combined organic extracts were passed through a phase separator and concentrated under reduced pressure. The resulting colourless oil was dissolved in petroleum ether (1 mL), followed by the addition of TFA (5 µL) and H_2_O (10 µL). The solution was purged with O_2_ and stirred at room temperature with an O_2_ balloon for two days. H_2_O (2 mL) and Et_2_O (2 mL) were added and the aqueous layer was extracted with Et_2_O (3 × 2 mL). The combined organic phases was dried over anhydrous Na_2_SO_4_ and concentrated under reduced pressure. The crude product was purified by flash chromatography on silica (ethyl acetate/hexane: 2:8 to 3:7) to give artemisinin (**1**) (<1 mg). (11*S*-**1**): ^1^H NMR (500 MHz, CDCl_3_): *δ* (ppm) 5.93 (1H, s), 3.70–3.67 (1H, m), 2.43–2.37 (1H, m), 2.29–2.25 (2H, m), 2.17–2.15 (2H, m), 2.08–2.05 (1H, m), 1.97–1.94 (1H, m), 1.82–1.79 (1H, m), 1.73–1.70 (1H, m), 1.64–1.63 (1H, m), 1.47 (3H, d, *J* = 8.1 Hz), 1.46 (3H, s) 1.11–1.19 (1H, m), 1.00 (3H, d, *J* = 5.4 Hz). ^13^C NMR (125 MHz, CDCl_3_): *δ* (ppm) 172.5, 105.3, 94.0, 77.2, 50.5, 45.5, 39.7, 37.6, 35.9, 34.0, 31.1, 25.5, 24.7, 20.5, 19.9. (11*R*-**1**): ^1^H NMR (500 MHz, CDCl_3_): *δ* (ppm) 5.86 (1H, s), 3.38–3.41 (1H, m), 2.44–2.40 (1H, m), 2.07–1.99 (2H, m), 1.89–1.88 (1H, m), 1.78–1.73 (2H, m), 1.52–1.35 (4H, m), 1.45 (3H, s), 1.21 (3H, d, *J* = 7.7 Hz), 1.02–1.13 (1H, m), 1.00 (3H, d, *J* = 5.4 Hz). HRMS calcd for C_15_H_23_O_5_ (M+H^+^) 283.1545, found 283.1559.
